# The Prevalence of Visual Acuity Impairment among School Children at Arada Subcity Primary Schools in Addis Ababa, Ethiopia

**DOI:** 10.1155/2017/9326108

**Published:** 2017-06-19

**Authors:** Haile Fentahun Darge, Getahun Shibru, Abiy Mulugeta, Yinebeb Mezgebu Dagnachew

**Affiliations:** ^1^Department of Medical Physiology, College of Medicine and Health Science, Bahir Dar University, Bahir Dar, Ethiopia; ^2^Department of Medical Physiology, College of Health Sciences, School of Medicine, Addis Ababa University, Addis Ababa, Ethiopia; ^3^Department of Ophthalmology, College of Health Sciences, School of Medicine, Addis Ababa University, Addis Ababa, Ethiopia

## Abstract

**Background:**

Visual impairment and blindness are major public health problems in developing countries where there is no enough health-care service.

**Objective:**

To determine the prevalence of visual impairment among school children.

**Materials and Methods:**

A school-based cross-sectional study was conducted between 15 June 2015 and 30 November 2015 at Arada subcity primary schools, Addis Ababa, Ethiopia. Two schools were selected randomly, and 378 students were screened from grades 1 to 8 using systematic random sampling method. Snellen chart was used for visual acuity test. Students who had visual acuity of ≤6/12 were further examined by an ophthalmologist to diagnose the reason for low vision. Data was analyzed using SPSS version 20.

**Results:**

A total of 378 students were screened, and 192 (50.8%) were females and the remaining 186 (49.2%) were males. The prevalence of visual impairment (VA) of ≤6/12 on either eye was 5.8%, VA < 6/18 on either eye was 1.1%, and VA < 6/18 on the better eye was 0.53%. In this study, color blindness [OR: 19.65, 95% CI (6.01–64.33)] was significantly associated with visual acuity impairment.

**Conclusion:**

The prevalence of visual impairment among school children in the study area was 5.8% and school screening is recommended.

## 1. Introduction

Visual system is one of our most important sensory systems. It is the primary means of integration between individuals and the external environments. Vision results from the entrance of light into the eye and the interpretation of this stimulus by the brain [1].

Low vision is defined as a visual acuity of less than 6/18, but equal to or better than 3/60, or a corresponding visual field loss of less than 20 degrees in the better eye with the best possible correction. On the other hand, blindness refers visual acuity of less than 3/60 or a corresponding field loss of less than 10 degrees in the better eye with the best possible correction [2]. It is estimated that 1.6 billion people in the world suffer from impaired visual acuity and the incidence is increasing [3].

It has been estimated that 75–90% of all learning in the classroom comes to the students either wholly or partially via the visual pathway [4]. Therefore, in children, visual impairment can affect school performance and other functions, such as ability to safely participate in sports. Poor performance at school may affect the child's self-confidence and their future careers. It has considerable social, psychological, and economic implications for the patients and their caregivers. Employment in certain professions like working in the capacity of pilots, drivers, and a few others also necessitates a normal vision and hence visually impaired persons are likely to be rejected from such professional jobs [5].

The major causes of low vision and blindness includes cataract, refractive error, and trachomatous corneal opacity. Major causes of low vision and blindness are either preventable or treatable [6–9]. According to WHO, about 285 million people are visually impaired worldwide and one individual becomes blind in each minute and a child in each 5 minutes. The burden of visual impairment is not distributed uniformly throughout the world. About 90% of visually impaired people are living in developing countries [10]. The poorest regions of Africa and Asia are where three quarters of the world's blind children live [11]. Out of the 1.4 million blind children globally, about 300,000 live in Africa. The prevalence of blindness in children in a country is related to the nutritional, health, and socioeconomic status of that country [11]. A study done on rural primary school children in Tanzania showed the prevalence of bilateral impaired visual acuity (VA < 6/12 in the better eye) of 0.7% [12].

Sub-Saharan Africa has an estimated 5-6 million blind and 16–18 million persons with low vision. Around 60% of them live in twenty African countries including Botswana, Eritrea, Ethiopia, Gambia, Ghana, Kenya, Lesotho, Liberia, Malawi, Mauritius, Namibia, Nigeria, Seychelles, Sierra Leone, South Africa, Swaziland, Uganda, the United Republic of Tanzania, Zambia, and Zimbabwe [13, 14].

The eye problem in Ethiopia is among the major public health challenges. It poses huge economic and social impacts for the affected individuals, the society, and the nation at large [15].

The prevalence of low vision in Ethiopia is 3.7% with considerable regional variations. The large proportion of this problem (91.2%) is due to avoidable (either preventable or treatable) causes [6, 16]. However, if it is not detected early, it may cause irreversible blindness. When visual loss is present at a young age, the adverse impact is felt over the remaining years of life.

Therefore, the main aim of this study was to determine the prevalence of visual acuity impairment among school children in Arada subcity, Addis Ababa, Ethiopia. It gives an evidence of the need of school screening for Ethiopia.

## 2. Materials and Methods

The study was conducted from June 10 to November 30, 2015 in two primary schools; the Holy Trinity Cathedral Primary School and the Zeray Deres Primary School in Arada subcity, Addis Ababa, Ethiopia. Two schools were selected randomly from 9 primary schools in the subcity, and 378 students were screened from grades 1 to 8 using systematic random sampling method. The total sample size was estimated using a single population proportion formula; the prevalence of low vision in Ethiopia (*P*) was taken to be 3.7% from the previous study [6], with a confidence interval of 95% and a marginal error of 2%. Contingency of 10% for the nonresponse rate was added.

Visual acuity was measured by using Snellen chart at 6 meters in a properly illuminated room. Each eye was tested separately three times, and the best was taken. Those who had low vision were further evaluated by an ophthalmologist to know the diagnosis and for proper management. The study subjects were considered to have low vision if their visual acuity was below 6/12 [2]. We have also used a structured questionnaire to assess the sociodemographic characteristics of the children and their parents.

Data analysis was carried out using SPSS version 20.0 for Windows. Bivariate logistic regression model was used to estimate the odds ratios (ORs) with their 95% confidence interval (CI) and to test the significant associations between the risk factors and visual acuity impairment. A *P* value of <0.05 was considered significant.

Ethical approval was obtained from Addis Ababa University Research and Ethical Review Board. Written and informed consent was obtained from parents or guardians of the children and confidentiality was maintained.

## 3. Results

A total of 378 students participated in the study. Among these, 255 (67.5%) were from the Holy Trinity Cathedral Primary School and 123 (32.5%) were from the Zeray Deres Primary School. Their age was from 5 to 16 years with mean age of 11.05 ± 2.58. One hundred sixty-two (42.9%) were from 1st to 4th grade and 216 (57.1%) were from 5th to 8th grade. The frequencies of females and males were 192 (50.8%) and 186 (49.2%), respectively (Table 1).

**Figure 1:**
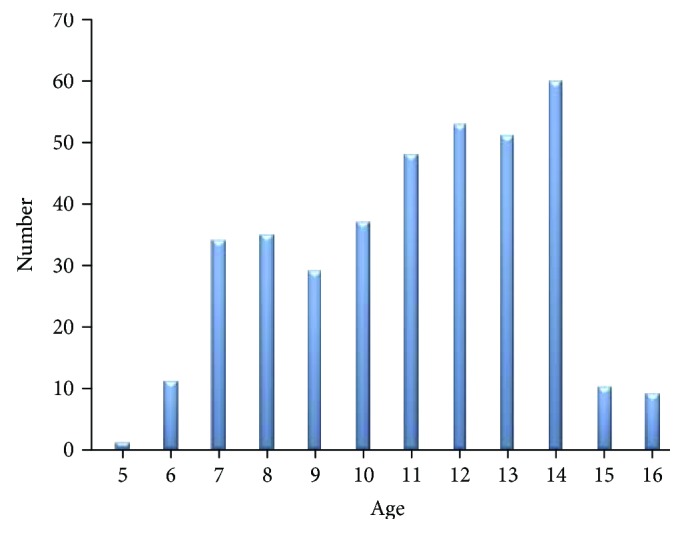
Distribution of participants by age.

Regarding age distribution, the minimum age was 5 and the maximum age was 16 years old. Majority of the participants, 60 (16%), were age of 14 (Figure 1).

With regard to educational level of the parents of the visually impaired students, only 4 (18%) had college diploma and above. In terms of income, large proportion, 19 (86.4%), of the parents of the visually impaired students have low income (≤2500 Ethiopian birr) (Table 2).

From the total participants, 22 (5.8%) were visually impaired (VA ≤ 6/12 in either eye) and 356 (94.2%) of them were normal (VA > 6/12 in the worse eye) (Table 3).

The frequencies of visually impaired female and male students were 12 (54.5%) and 10 (45.5%), respectively (Table 3). However, sex was not statistically significant with visual impairment (*P* = 0.38) (Table 4). Among the visually impaired students, 6 (27.3%) were from 5 to 8 years old, 9 (40.9%) were from 9 to 12 years old, and 7 (31.8%) were from 13 to 16 years old (Table 3).

Color vision (OR = 19.65; 95% CI: 6.01–64.33) and low grade level (OR = 0.12; 95% CI: 0.03–0.58) were significantly associated with visual acuity impairment (Table 4).

As per the ICD-9 guideline, the study showed that 5.8% of the total participants had visual acuity impairment (Table 5).

From the total visually impaired children, 18 (4.7%) had visual acuity of 6/12–6/18 and all of them had bilateral visual problem. The remaining 4 (1%) had visual acuity of <6/18–6/60. Two (0.5%) of them had bilateral visual problem, and the remaining 2 (0.5%) had unilateral visual impairment (Table 6).

**Figure 2:**
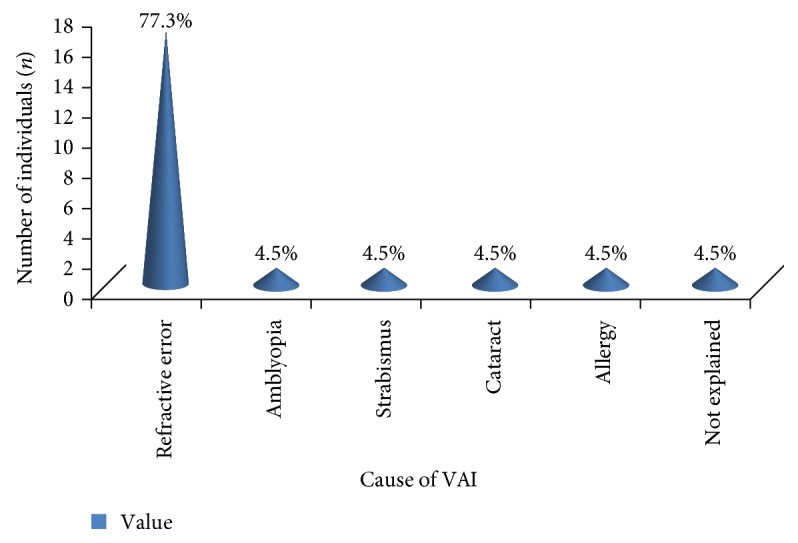
Cause of visual acuity impairment.

Visual acuity impairment was caused by different factors. Refractive error was the leading cause of visual impairment in this study which accounts for 17/22 (77.3%) of the causes. Cataract, severe allergy, amblyopia, and strabismus each accounts for 1/22 (4.5%) of the causes. The cause of abnormal vision of one student was not known because he was absent at the time of examination by an ophthalmologist (Figure 2).

## 4. Discussion

According to this study, the prevalence of visual impairment VA ≤ 6/12 in either eye was 22 (5.8%), VA < 6/18 in either eye was 4 (1.1%), and VA < 6/18 in the better eye was 2 (0.53%). This finding is less than the study done nationally in Ethiopia which was 3.7% [6, 15]. The low prevalence rate of this study might be because of the difference in study design and area. This study was conducted on elementary students in Addis Ababa, the capital city of Ethiopia, where eye health services are available and awareness is good. The other study was community based and involved every part of Ethiopia and all age groups were included. As age increases, the prevalence of visual acuity impairment will increase [17, 18].

The study done in Malaysia and Indonesia [17, 19] showed that reduction in visual acuity had a linear relationship with increasing age. However, in our study, as shown in Table 3, 9 (2.4%) of children from 9 to 12 years old had VA ≤ 6/12 in either eye which is greater than children aged 5–8 and 13–16 years old that accounts for 6 (1.6%) and 7 (1.9%), respectively. This indicates that there was no statistically significant association between age and visual acuity impairment (OR = 4.57, 2.73, *P* > 0.05). This may be due to the fact that in this study, the sample size comprised of a small age difference which was between 5 and 16 years old with mean age 11.05 ± 2.58; therefore, the association between age and reduction of visual acuity may not be seen. The result is in line with another study done in Malaysia which states that age factor was not associated with reduction of visual acuity [20].

In this study, the prevalence of visual acuity impairment was slightly higher among females (3.2%) as compared to males (2.6%). This is supported by the study done in India [22]. This might be due to socioeconomic factors that contribute to access to health services. However, the difference was not statistically significant (OR = 0.65, *P* = 0.38).

Different studies showed a significant association between visual acuity impairment in children with income, educational background, and visual status of parents. A study done in South Africa [21] showed that poor protein, fruit, and vegetable intake led to poor visual acuity in the subjects. In our study, the income of majority of the parents of the visually impaired children, 8 (36.4%), were from 1501 to 2500 birrs/month and only 3 (13.6%) had more than 2500 birrs/month. This shows that the incomes of most parents are low and their children might not get a balanced diet and this may contribute to poor visual acuity.

Regarding educational background of parents, only 4 (18.1%) had college diploma and university degree. More than 81% of them had no higher education. Studies done in India, China, and Nepal reported that visual problems were found around three times more in those who have no schooling than those who have schooling [21, 23, 24].

In our study, almost half of the parents of the visually impaired children had visual problems including myopia, hyperopia, color blindness, and undefined blindness. Causes of low vision like refractive errors can be inherited. A study done by Mutti et al. stated that the risk of inheriting impaired visual acuity was increased if parents had similar problems [25].

In this study, 31.8% of individuals who had VA ≤ 6/12 had color vision defect. There was a strong association between visual acuity impairment and color blindness (OR = 19.65, *P* < 0.01). This implies that being color vision defective is more at risk to individuals with visual impairment than to individuals who had normal color vision. This is due to the fact that missing, dead, or damaged cone cells result in loss of both acuity and color perception at the same time [26]. Another study done by Delpero et al. suggested that acquired color vision defect may escape detection but, if severe, is also associated with loss of visual acuity and/or visual field [27].

A study done in India [28], Malaysia [29], and China [30] and a study done in African children [31] suggested that uncorrected refractive error as the main cause of visual impairment. Our study also showed that uncorrected refractive error was the main cause for defective vision. Seventeen (77.3%) of the visually impaired students had refractive error. It is consistent with another study done in India which states that refractive error accounts for 77% of the total cause of visual impairments [32, 33].

## 5. Conclusion and Recommendation

The prevalence of visual impairment in two elementary schools in Addis Ababa showed that VA ≤ 6/12 in either eye was 22 (5.8%), VA < 6/18 in either eye was 4 (1.1%), and VA < 6/18 in the better eye was 2 (0.53%). School screening is recommended for early detection and possible management.

## Figures and Tables

**Table 1:** Distribution of participants by grade, school, and sex.

Grade	Primary schools	Sex		Total
		F	M	
1st–4th	The Holy Trinity Cathedral	48 (12.7%)	53 (14.0%)	101 (26.7%)
	Zeray Deres	29 (7.7%)	32 (8.5%)	61 (16.2)
	Total	77 (20.4%)	85 (22.5%)	162 (42.9%)
5th–8th	The Holy Trinity Cathedral	86 (22.8%)	68 (18.0%)	154 (40.7%)
	Zeray Deres	29 (7.7%)	33 (8.7%)	62 (16.4%)
	Total	115 (30.4%)	101 (26.7%)	216 (57.1%)
Total	The Holy Trinity Cathedral	134 (35.4%)	121 (32.0%)	255 (67.5%)
	Zeray Deres	59 (15.7%)	64 (16.9%)	123 (32.5%)
	Grand total	192 (50.8%)	186 (49.2%)	378 (100%)

**Table 2:** Sociodemographic features of parents of students who have VA ≤ 6/12.

*Age*	*N* (%)
25–40	15 (68.2%)
41–55	4 (18.2%)
56–70	3 (13.6%)
*Educational background*	
Illiterate	1 (4.5%)
Elementary school	5 (22.7%)
Secondary school	12 (54.5%)
College diploma	3 (13.6%)
University degree	1 (4.5%)
*Income*/*month* (*Ethiopian birr*)	
150–800	5 (22.7%)
801–1500	6 (27.3%)
1501–2500	8 (36.4%)
2501–3500	3 (13.6%)
*Visual status*	
Blind	1 (4.5%)
Color blind	1 (4.5%)
Hyperopia	1 (4.5%)
Myopia	7 (31.8%)
No eye problem	12 (54.5%)
*Eye care for their child*
Washing with soap	11 (50%)
No care for the eye of their child	11 (50%)

**Table 3:** The frequency of visual impairment (VA ≤ 6/12) by sex and schools.

		Primary schools			
		Holy Trinity Cathedral (*n* = 22)	Zeray Deres (*n* = 22)	Total (*n* = 22)	% (*n* = 378)
Sex	F	8 (36.4%)	4 (18.2%)	12 (54.5%)	3.2
	M	6 (27.3%)	4 (18.2%)	10 (45.5%)	2.6
	Total	14 (63.7%)	8 (36.4%)	22 (100.0%)	5.8
Age	5–8	5 (22.7%)	1 (4.5%)	6 (27.3%)	1.6
	9–12	6 (27.3%)	3 (13.6%)	9 (40.9%)	2.4
	13–16	3 (13.6%)	4 (18.2%)	7 (31.8%)	1.9
	Total	14 (63.7%)	8 (36.4%)	22 (100%)	5.8

**Table 4:** Bivariate logistic regression analysis of factors associated with VAI.

Variables	Visual acuity				Total		OR (95% CI)	*P* value
	VA > 6/12		VA ≤6/12					
	*n*	%	*n*	%	*n*	%		
*Sex*								
Female	180	47.6	12	3.2	192	50.8	1	0.38
Male	176	46.6	10	2.6	186	49.2	0.65 (0.25–0.67)	
*Age*								
5–8	75	19.8	6	1.6	81	21,4	1	0.26
9–12	158	41.8	9	2.4	167	44.2	4.57 (0.74–28.34)	0.10
13–16	123	32.5	7	1.9	130	34.4	2.73 (0.58–12.964)	0.19
*School*								
HTCPS	241	63.8	14	3.7	255	67.5	1	0.74
ZDPS	115	30.4	8	2.1	123	32.5	0.84 (0.30–2.34)	
*Grade*								
1–4	147	38.9	15	4.0	162	42.9	1	0.01^∗^
5–8	209	55.3	7	1.9	216	57.1	0.12 (0.03–0.58)	
*Color vision*								
Normal	347	91.8	15	4.0	362	95.8	1	0.00^∗^
Defective	9	2.4	7	1.9	16	4.2	19.65 (6.01–64.33)	

*Note*. ^∗^Statistically significant at 95% CI, *P* < 0.05; 1: reference; HTCPS: Holy Trinity Cathedral Primary School; ZDPS: Zeray Deres Primary School; VA: visual acuity; VAI: visual acuity impairment.

**Table 5:** Frequency of visual acuity with the worse eye.

VA of the worse eye		ICD-9, 10-CM categories	WHO	Age group (in years)			Total	% out of total (*n* = 378)	% (*n* = 378)
				5–8	9–12	13–16			
VAI	6/60	Sever VI	Low vision	—	1	—	1	0.3%	1.1%
	6/36	Moderate VI		1	2	—	3	0.8%	
	6/18	Mild VI	Normal vision	1	2	2	5	1.3%	98.9%
	6/12	Mild V I		4	4	5	13	3.4%	
Total				**6**	**9**	**7**	**22**	**5.8%**	
Normal	6/6	Normal		75	158	123	356	94.2%	
Grand total				**81**	**167**	**130**	**378**	**100%**	**100%**

VI: visual impairment; VA: visual acuity; VAI: visual acuity impairment.

**Table 6:** Distribution of VAI by sex in one and both eyes.

VA	Sex		Total (*n* = 378)
	Male	Female	
*≤6/12 to ≥6/18*			
Bilateral	8 (2.1%)	10 (2.6%)	18 (4.7%)
Unilateral	0 (0%)	0 (0%)	0 (0%)
*<6/18 to ≥6/60*			
Bilateral	1 (0.3%)	1 (0.3%)	2 (0.5%)
Unilateral	1 (0.3%)	1 (0.3%)	2 (0.5%)
*Total*	**10 (2.6%)**	**12 (3.2%)**	**22 (5.8%)**

VA: visual acuity.
